# Nanoscale Graphene Disk: A Natural Functionally Graded Material–How is Fourier’s Law Violated along Radius Direction of 2D Disk

**DOI:** 10.1038/srep14878

**Published:** 2015-10-07

**Authors:** Nuo Yang, Shiqian Hu, Dengke Ma, Tingyu Lu, Baowen Li

**Affiliations:** 1 Nano Interface Center for Energy (NICE), School of Energy and Power Engineering, Huazhong University of Science and Technology (HUST), Wuhan 430074, People’s Republic of China; 2 State Key Laboratory of Coal Combustion, Huazhong University of Science and Technology (HUST), Wuhan 430074, People’s Republic of China; 3 Center for Phononics and Thermal Energy Science, School of Physics Science and Engineering, Tongji University, 200092 Shanghai, People’s Republic of China; 4 Department of Mechanical Engineering, University of Colorado, Boulder, CO 80309

## Abstract

In this Paper, we investigate numerically and analytically the thermal conductivity of nanoscale graphene disks (NGDs), and discussed the possibility to realize functionally graded material (FGM) with only one material, NGDs. Different from previous studies on divergence/non-diffusive of thermal conductivity in nano-structures with different size, we found a novel non-homogeneous (graded) thermal conductivity along the radius direction in a single nano-disk structure. We found that, instead of a constant value, the NGD has a graded thermal conductivity along the radius direction. That is, Fourier’s law of heat conduction is not valid in two dimensional graphene disk structures Moreover, we show the dependent of NGDs’ thermal conductivity on radius and temperature. Our study might inspire experimentalists to develop NGD based versatile FGMs, improve understanding of the heat removal of hot spots on chips, and enhance thermoelectric energy conversion efficiency by two dimensional disk with a graded thermal conductivity.

The novel properties of nanostructures attracted many attentions recently. For example, the Fourier’s law, govern heat conduction in macroscopic, is not valid in one dimensional nanostructures^1^,^2^,^3^,^4^,^5^,^6^, due to the confinement effect. It is shown that the thermal conductivity depends on the length (size) of the nanostructure.

For two dimensional structure graphene^7^, its thermal properties have attracted immense interests recently^8^, because the size dependence^9^,^10^ and the super-high value^11^,^12^ of thermal conductivity have been observed. Because phonons (lattice vibrations) dominate thermal transport in graphene, it raised the exciting prospect to use graphene-based nano-sheets as thermal (phononics) devices^13^,^14^,^15^,^16^. Besides, the management of phonons provide advances in thermal devices, such as thermal diodes^17^, thermal cloaking^18^,^19^, thermoelectrics^20^, and thermocrystals^21^,^22^,^23^.

Previous studies^9^,^12^,^15^,^24^ focused on the divergence of thermal conductivity along the longitudinal directions by keeping the width of transverse direction fixed. In contrast, we focus on the heat transfer along the radius direction and find the novel property, a graded thermal conductivity. That is, the graphene disk is not homogeneous along the radius direction. Both the previous simulation^25^ and measurements of a 2D nanoscale disk^11^,^26^,^27^ assumed that the thermal conductivity is a constant along the radius direction. That is, the graphene disk is a natural graded structure in thermal conductivity.

A functionally graded material (FGM) is a composite, consisting of two or more phases, which is fabricated such that its composition varies in a certain spatial direction^28^,^29^. The concept of FGMs was proposed in 1984 by material scientists as a means of preparing thermal barrier materials^30^. This design is intended to take advantage of certain desirable features of each of the constituent phases. Recently, another attempt to apply the FGM concept to the enhancement of thermoelectric (TE) energy conversion efficiency has been initiated^31^,^32^,^33^.

TE materials are important for generating electricity from waste heat and being used as solid-state Peltier coolers. The performance of thermoelectric materials depends on the figure of merit (ZT). A functional graded thermoelectric material (FGTEM) can maintain high values of ZT over a wide range of temperatures, for example, by controlling a gradual change of dopant concentration along the length of a TE device. Muller *et al.* reviewed various methods for fabrication of FGTEMs and how the gradient impacts the resulting efficiencies^32^.

In this paper, we investigate the thermal conductivity of nanoscale graphene disks (NGDs) by using molecular dynamics (MD) and analytical analysis, and discussed the possibility to realize FGM with only one material, NGDs. A NGD structure is shown in the inset of Fig. 1, where the difference between inside radius (r_in_) and outside radius (r_out_) is defined as L. The lattice constant (a) and thickness (d) of NGD are 0.1418 nm and 0.334 nm, respectively. Moreover, we show the dependence of NGDs’ thermal conductivity on radius and temperature.

## Numerical and analytical results

**Figure 1:**
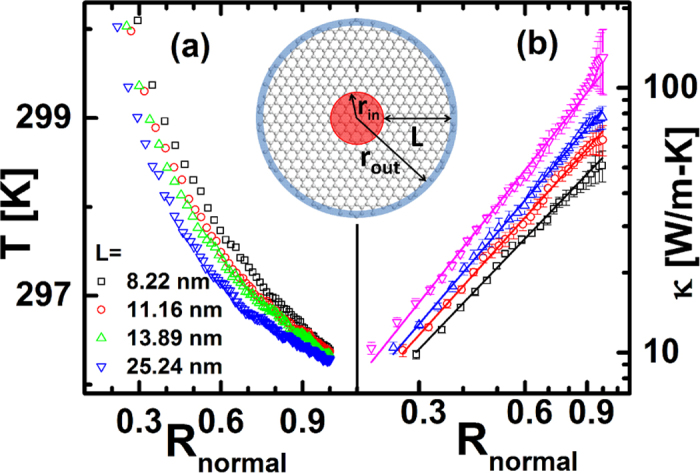
(**a**) Temperature profiles along the radial direction with different outer radius (r_out_) at 300 K. The normalized radius, R_normal_, is defined in Eq. (2). (**b**) The thermal conductivity of graphene disk with different r_out_ at 300 K. The symbols are numerical data and the lines are fitted lines. The fitted values of *α* are 1.38 ± 0.03, 1.47 ± 0.01, 1.55 ± 0.01, 1.66 ± 0.02 corresponding to r_out_ as 8.22, 11.16, 13.89, and 25.24 nm, respectively. The error bar is a combination of the error in the calculation of temperature gradient and the standard error of heat flux. The inset is schematic picture of graphene disk structure. The inside atoms (shaded in red) and the outside atoms (shaded in blue) are controlled by heat baths with temperatures T_in_ and T_out_, respectively.

For a homogeneous disk of bulk material, the thermal conductivity (*κ*) is defined as


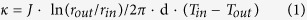


where J is the heat current, d is the thickness of NGD. However, in the calculation of *κ* of a non-homogeneous structure, the temperature gradient, *dT/d*1n(*r*), is an important factor. As shown in Fig. 1(a), different from bulk disk structures, the temperature gradient of NGDs is not a constant and depends on radius r. The values of temperature gradient for different r are obtained directly from the discrete temperature profiles of MD. Then, we calculated the NGD’s thermal conductivity with different outer radius at room temperature shown in Fig. 1(b). It is obviously that the values of thermal conductivity of each NGD are not constants, instead, it is dependent on the radius. The closer the atoms to the disk center, the smaller the thermal conductivity. That is, the NGD is a special structure with a graded thermal conductivity.

In the above, we obtained the discrete data of temperature profiles in NGD by MD simulations, and calculated the temperature gradients and thermal conductivities directly from the raw discrete MD data. However, there are large errors in thermal conductivity, especially for larger r, due to the fluctuation of temperature profiles.

As the curves of *κ* on a log-log plot are linear (Fig. 1(b)), we can write:


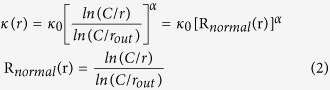


where *κ*_0_ and C are constants, and *α* is power-law exponent fitted from MD results.

Obviously, Fourier’s law is not valid in this system and it is familiar that the power law size dependence of thermal conductivity. In the past decade, some results have revealed the breakdown of Fourier’s law in low dimensional system. It was found that a superdiffusive thermal transports and a power law length dependent of thermal conductivity in both carbon nanotubes and silicon nanowires^13^. Recently, Vermeersch *et al.* also found and explained the superdiffusive motion of thermal energy in semiconductor alloys theoretically^34^,^35^. All the previous studies reveal a homogeneous thermal conductivity. Here, the graded thermal conductivity along the radius direction is a new phenomenon. When the disk is treated as a serial of rings with different radius and perimeters, the rings have a power law length dependent of thermal conductivity.

For the simplicity, we define the normalized radius, R_normal_, as a function of r. Similar to the solutions in a hollow circular cylinder FGM^29^, we derive the analytical results of non-homogeneous steady state temperature distributions in NGDs (the detailed derivations are given in the Supporting information II). The solutions are:


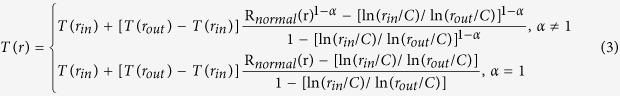


where, the value of C is chosen as r_in_/e for the purpose of normalization in radius.

**Figure 2:**
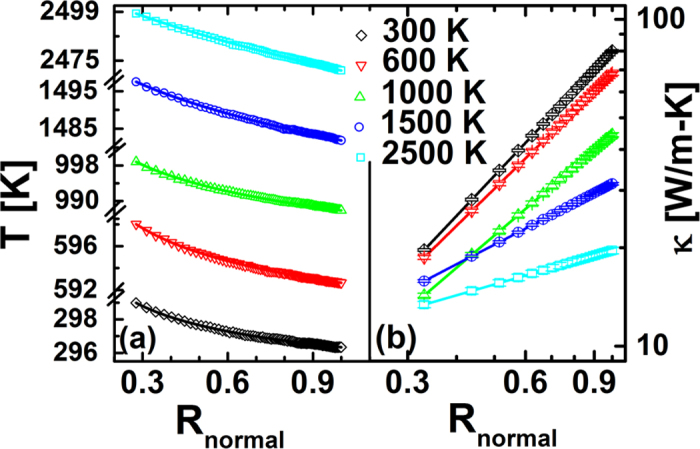
(**a**) The temperature profiles of nanoscale graphene disk (NGD) along the radial direction with different temperatures. The normalized radius, R_normal_, is defined in Eq. (2). The outer radius of NGD equals 13.89 nm. The symbols are MD simulation results and the lines are fitted lines based on our analytical results, Eq. (3). The fitting values of *α* are 1.26 ± 0.01, 1.17 ± 0.01, 1.02 ± 0.01, 0.62 ± 0.01, 0.34 ± 0.01 corresponding to the temperature as 300, 600, 1000, 1500, 2500 K, respectively. (**b**) The thermal conductivity of graphene disk with different temperature. The graded thermal conductivities have form as shown in Eq. (1). The error bar is a combination of the error in the calculation of temperature gradient and the standard error of heat flux (an average of 12 MD simulations with different initial conditions).

**Figure 3:**
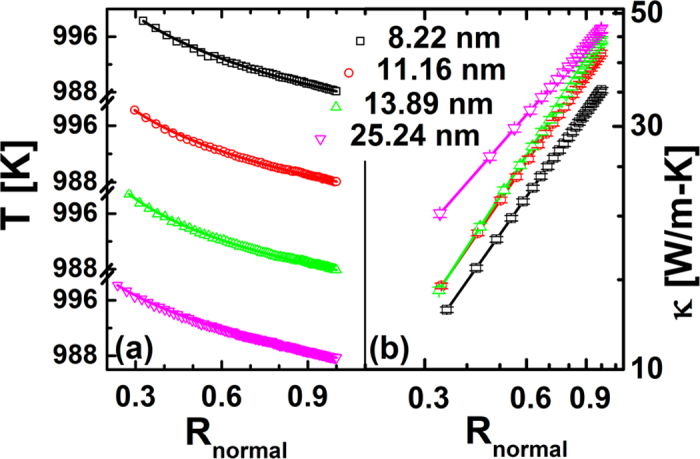
(**a**) The temperature profiles along the radial direction with different outer radius (r_out_) at 1000 K. The symbols are numerical data and the lines are fitted lines based on our analytical results, Eq. (3). The fitting values of *α* are 0.94 ± 0.02, 0.97 ± 0.01, 1.02 ± 0.01, 0.76 ± 0.01 corresponding to r_out_ as 8.22, 11.06, 13.89, 25.24 nm, respectively. (**b**) The thermal conductivity of graphene disk with different r_out_ at 1000 K. The error bar is a combination of the error in the calculation of temperature gradient and the standard error of heat flux.

Now, we can use Eq. (3) to fit the temperature profile data by MD, which may overcome the problem of fluctuation in temperature profile. In the following, different from results in Fig. 1, the thermal conductivities in Fig. 2 and 3 will be calculated by the fitting curves based on Eq. (3), which has smaller error than results in Fig. 1.

Then, we investigate the temperature and radius effect on the thermal conductivity of NGD by MD simulations. Firstly, we choose a disk with the outer radius as 13.89 nm and calculate the thermal conductivity of different temperatures from 300 K to 2500 K.

The temperature profiles along the radial direction are plotted in Fig. 2(a). It is shown that the numerical data can be well fitted by our analytical results in Eq. (3), which give evidence of our argument of graded thermal conductivity in Eq. (2). The fitted temperature gradient has a much smaller fluctuation comparing to the data extracted directed from the discrete temperature profiles of MD. The fitted values of *α* decreased toward zero as the temperature increasing. That is, for the high temperature limit, the temperature profile in NGD would be close to linear and there would be no graded thermal conductivity.

As shown in Fig. 2(b), the thermal conductivities of NGD are calculated with different temperatures from 300 K to 2500 K. When the temperature is 300 K, the value of thermal conductivity of the outermost ring is nearly four times larger than the value of innermost ring. As the temperature increases to 2500 K, the value of *κ*(*r*_*out*_) is almost as twice as that of *κ*(*r*_*in*_). It is clear shown that the NGD structures have graded thermal conductivity and can be used as FGM in a large temperature range, which is the main result of this paper. Additionally, due to the different ways in calculating temperature gradients, the value of *α* at 300 K in Fig. 2, is a little different from that in Fig. 1 for 13.89 nm NGD, 1.55.

Besides the temperature effect, we also investigate the size effect on thermal conductivity of NGD at 1000 K, where the *r*_*out*_ are changed from 8.22 to 25.24 nm. The temperature profiles along the radial direction with different outer radius are shown in Fig. 3(a), where the temperature profile by MD can be well fitted by our analytical results in Eq. (3). The thermal conductivity of NGDs with the different r_out_ are calculated and plotted in Fig. 3(b). The results show that the values of *α* is not sensitive of *r*_*out*_. However, the values *κ*_0_ depends on the *r*_*out*_. That is, the value of thermal conductivity is enhanced with the increase of NGD’s outer radius.

## Discussions and Conclusions

Based on our MD simulation results of the NGD’s thermal conductivity (*κ*), we found that *κ*(*r*) depends on radius and increase gradually with r as shown in Eq. (2), 
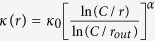
. The value of α would depend on temperature and approach to zero at high temperature limit. That is, there would be less graded effect in NGDs in the high temperature limit where the nonlinear effects are dominant and there are many more phonon-phonon couplings. The value of *κ*_0_ depends on the geometric size of NGDs when temperature is fixed. The larger the outer radius of NGD, the higher the value of *κ*_0_. The trend is in consistent with the phonon transport in nanostructures^13^, where there are larger mean free path and more eigenmodes in a larger structure.

Recently, the size and confinement effect in thermal properties of nanostructures are well studied^5^,^6^,^36^,^37^.

**Figure 4:**
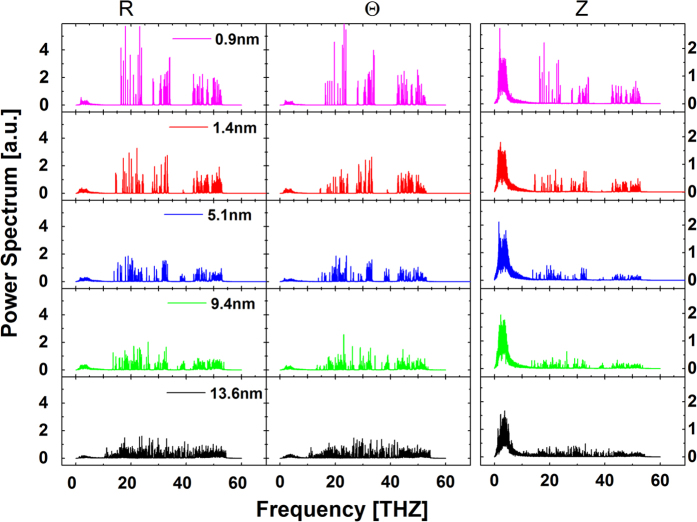
Normalized power spectra of graphene rings along two in-plane directions (r and θ) and out-plane direction (z). The radiuses of rings are 0.9, 1.4, 5.1, 9.4, and 13.6 nm, respectively.

The thermal conductivity of nanostructures depends on the size^3^,^4^,^38^,^39^ and the Fourier’ law is not valid in nanoscale. A nanoscale graphene disk can be looked as a serial of thin graphene rings with different radius from r_in_ to r_out_. We calculated the power spectra of several rings with different radius in Fig. 4, to understand the underlying mechanism in nanoscale. The spectra are obtained by Fourier transforming velocity autocorrelation function.

Due to the finiteness of size, the phonon wavelength in a ring is limited from the lattice constant to the circumference, which leads to the confinement in phonon’s modes shown in the spectra of nanoscale rings obviously. That is, the number of peaks, which corresponding to egienmodes, is much decreased as the radius is decreased. Analogy to nanowires and nanotubes with different length^3^,^4^,^38^,^39^, the ring with different radius will have different ability in heat transfer. For a smaller ring, the thermal conductivity will be lower due to the very small number of egienmodes. With the increase of radius, more and more phonons, especially phonons with longer wavelengths, are existed in the ring, which will result in the increase of thermal conductivity. When the rings are connected one by one, the structure is nonhomogenous along the direction of thermal gradient, and a graded structure will be built.

**Figure 5:**
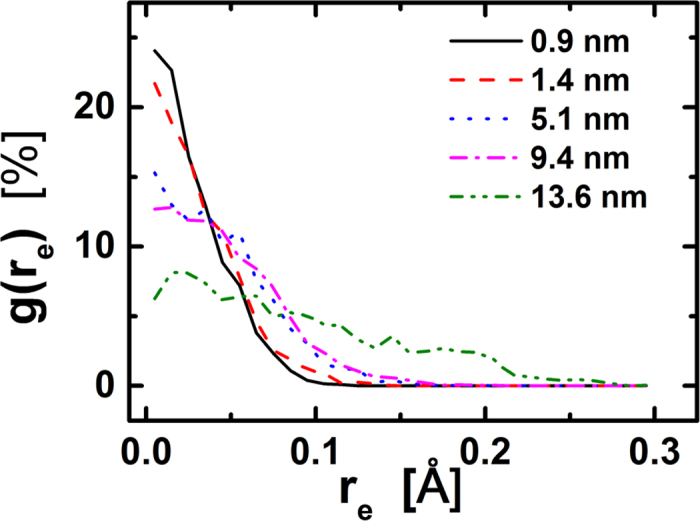
The atomic position density profiles around its equilibrium positions, g(r_e_), in an entire graphene disk, where r_e_ is the distance to the atom equilibrium position. There is obvious difference in g(r_e_) for atoms with different distance to the disk center.

We recorded atom trajectories of vibration and calculated the atomic position density profiles, g(r_e_), within an entire graphene disk (shown in Fig. 5). It is found that there is obvious difference in g(r_e_) for atoms with different distance to the disk center. The atoms closer to the center are confined in a small region around its equilibrium position. On the contrary, the atoms far away from the center have a large spread of vibrations which corresponds to a combination of more phonon modes and a high thermal conductivity.

In summary, by using the classical nonequilibrium MD method, we have investigated the radius and temperature effect on the thermal conductivity of NGD. Interestingly, the Fourier’s law is not valid in NGD which is not homogeneous in thermal conductivity. It is found that the values of thermal conductivity of NGD are dependent on the radius. The closer the atoms to the disk center, the smaller the thermal conductivity. Namely, the NGD is a natural structure with a graded thermal conductivity, without any artificial compounding. Due to the low thermal conductivity close to the center, it may explain the difficulty of heat removal of hot spots on chips. Moreover, the thermal property of NGDs can be modulated by adjusting the temperature and the size. On the other hand, our analytical results of the temperature profile in NGD, Eq. (3), can fit simulation results very well. It is shown that our prediction of thermal conductivity in NGD, Eq. (2), is reasonable. Our study may inspire experimentalists to develop NGD based versatile FGMs, deepen the understanding the heat removal of hot spots on chips, and enhance thermoelectric energy conversion efficiency by NGD with a graded thermal conductivity.

Due to the limitation of hardware, the largest radius in simulations is 25 nm which is smaller than the mean free paths in graphene (~775 nm)^40^. It is still an open question for future study whether the NGD with a radius close to/larger than mean free path has a graded thermal conductivity.

## MD Simulation Methods

As we study the thermal conductivity of NGD by using classical non-equilibrium MD (NEMD) method, a temperature gradient is built in NGD along the radial direction. The NGD can be considered as a serial rings (1^st^, 2^nd^,…,N^th^) whose thickness is defined as a. In order to establish a temperature gradient, the atoms from the 2^nd^ to 4^th^ rings and the atoms in the (N-1)^th^ ring are controlled by Nosé-Hoover heat baths^13^ with temperatures T_in_ and T_out_, respectively. In some low dimensional structures, Nosé-Hoover heat baths is not sufficiently chaotic^41^. To ensure our results are independent of heat bath, Langevin heat bathes^42^ are also used. The results by both types of heat baths give rise to the graded thermal conductivity (details of results by Langevin in supporting information I). The atoms at boundaries (the 1^st^ and N^th^ rings) are fixed.

The potential energy is described by a Morse bond and a harmonic cosine angle for bonding interaction, which include both two-body and three-body potential terms^43^,^44^. Although this force field potential is developed by fitting experimental parameters for graphite, it has been testified by the calculation of thermal conductivity of carbon nanotubes^45^. To integrate the discretized differential equations of motions, the velocity Verlet algorithm is used. The MD simulation time step, Δt, is chosen as 0.5 fs.

Simulations are carried out long enough (2 ns) to guarantee that the system would reach a steady state. Then, the kinetic temperature (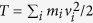) at each ring and the heat flux in each thermal bath are averaged over 3 ns.

The heat flux (J) transferred across the each ring can be calculated at the heat bath region as


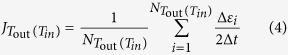


where Δε is the energy added to/removed from each heat bath (T_in_ or T_out_) at each step Δt. The thermal conductivity (κ) are calculated based on the Fourier definition as


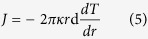


where r is the radius of each ring. We use a combination of time and ensemble sampling to obtain better average statistics. The results represent averages from 12 independent simulations with different initial conditions. Each case runs longer than 3 ns after the system reached the steady state.

## Additional Information

**How to cite this article**: Yang, N. *et al.* Nanoscale Graphene Disk: A Natural Functionally Graded Material-How is Fourier's Law Violated along Radius Direction of 2D Disk. *Sci. Rep.* **5**, 14878; doi: 10.1038/srep14878 (2015).

## Supplementary Material

Supporting Information
