# Supplementary material for: Nanoscale Graphene Disk: A Natural Functionally Graded Material–How is Fourier’s Law Violated along Radius Direction of 2D Disk
Source: Sci Rep. 2015 Oct 7;5:14878. doi: 10.1038/srep14878 (PMC4595637; doi:10.1038/srep14878)
Supplement: Supporting Information [file srep14878-s1.doc]

Supporting Information

**Nanoscale Graphene Disk: A Natural Functionally Graded Material**

**--** How is Fourier’s Law Violated along Radius Direction of 2D Disk

Nuo Yang^1,2,#,*^, Shiqian Hu^3,#^, Dengke Ma^1^, Tingyu Lu^3^, and Baowen Li^3,4*^

^1^ Nano Interface Center for Energy (NICE), School of Energy and Power Engineering, Huazhong University of Science and Technology (HUST), Wuhan 430074, People's Republic of China

^2^ State Key Laboratory of Coal Combustion, Huazhong University of Science and Technology (HUST), Wuhan 430074, People's Republic of China

^3^ Center for Phononics and Thermal Energy Science, School of Physics Science and Engineering, Tongji University, 200092 Shanghai, People's Republic of China

^4^ Department of Mechanical Engineering, University of Colorado, Boulder, CO 80309

^#^ N. Yang and S. Hu contributed equally to this work.

^*^ Corresponding authors:

Email: nuo@hust.edu.cn (N.Y.)

Email: phononics@tongji.edu.cn (B.L.)

**I. Molecular dynamics results using Langevin heat bathes**

The Nanoscale Graphene Disk (NGD) can be look as a serial rings (1^st^, 2^nd^,…,N^th^) whose thickness is defined as a. In order to establish a temperature gradient, the atoms from the 2^nd^ to 4^th^ rings and the atoms in the (N-1)^th^ ring are controlled by heat baths with temperatures T_in_ and T_out_, respectively. To ensure our results are independent of heat bath, Langevin heat bathes are also used. The results by both Nosé-Hoover and Langevin heat baths give rise to the graded thermal conductivity.

The temperature profiles along the radial direction are plotted in figure S1(a). It is shown that the numerical data can be well fitted by our analytical results in Eq. (S15), which give evidence of our assumption of graded thermal conductivity in Eq. (S1).

As shown in figure S1(b), the thermal conductivities of NGD are calculated with different temperatures from 300 K to 2500 K. When the temperature is 300 K, the value of thermal conductivity of the outermost ring is nearly four times larger than the value of innermost ring. As the temperature increases to 2500 K, the value of $\kappa\left(r_{out}\right)$ is almost as twice as that of $\kappa\left(r_{in}\right)$. It is clear shown that the NGD structures have graded thermal conductivity and can be used as FGM in a large temperature range, which is the main result of this paper.

**II. Derivation processes of non-homogeneous steady state temperature distributions in nanoscale graphene disks (NGDs)**

We considered a hollow NGD with an inner radius $r_{in}$, outer radius $r_{out}$, and thermal conductivity:

$\begin{matrix} \kappa(r)=\kappa_{0}\left[\frac{\ln(C/r)}{\ln(C/r_{out})}\right]^{\alpha}=\kappa_{0}\text{R}_{normal}\left(\text{r}\right)^{\alpha} \\ \begin{matrix} & & \end{matrix}\text{R}_{normal}\left(\text{r}\right)=\frac{\ln(C/r)}{\ln(C/r_{out})} \end{matrix}$ (S1)

where $\alpha$ would depend on temperature and geometric size of NGDs, $\kappa_{0}$ and C are constants. A cylindrical coordinate system $(r,\theta)$ is established for reference.

At the beginning, we consider the temperature of axial symmetry. For the special case of $\alpha=0$, i.e. when the material is homogeneous, the temperature solution is

$T(r)=F+G(-\ln r+\ln C)$ (S2)

where F and G are constants, depending on the boundary conditions of the problem.

To obtain the temperature solution for the non-homogeneous NGD, we proceed as follows:

1. Section the graphene rings (GRs) by radius where $r_{1}=r_{in}+h$, $r_{2}=r_{1}+h=r_{in}+2h$, etc., to divide the whole disk into N sub-rings of uniform thickness$h=(r_{out}-r_{in})/N$. So, the innermost sub-ring is numbered as sub-ring 1, the sub-ring next to it as sub-ring 2, and so on.
2. Assume that the non-homogeneous sub-rings are homogeneous, with a constant conductivity$\kappa_{0}\left[\frac{\ln(C/r_{j-1})}{\ln(C/r_{out})}\right]^{\alpha}$, where $r_{j-1}=r_{in}+(j-1)h$, corresponding to the ^th^ sub-ring.

The solution in Eq. (S2) now applies to each of the sub-rings, which have been assumed to be approximately homogeneous. For the j^th^ sub-ring, the temperature is denoted as

$T^{(j)}(r)=F^{(j)}+G^{(j)}(-\ln r_{j-1}+\ln C)$ (S3)

where the superscript indicates that the affixed quantity belongs to the j^th^ sub-ring.

1. For a large N, the thickness is small and the difference between and should be insignificant. The combination of solutions (S3), , should be a good approximation of the solution of the NGD, provided that the constants are determined first by the continuity conditions of temperatures and heat flux at the interfaces of the sub-rings, and finally by the boundary conditions of the NGD.

The continuity conditions of the temperatures and heat flux at $r=r_{1}$, which is the interface of the two innermost sub-rings, take the following form:

$\begin{matrix} F^{(2)}+G^{(2)}(-\ln r_{1}+\ln C)=F^{(1)}+G^{(1)}(-\ln r_{1}+\ln C) \\ \kappa^{(2)}\frac{G^{(2)}}{r_{1}}=\kappa^{(1)}\frac{G^{(1)}}{r_{1}} \end{matrix}$ (S4)

Eq. (S4) can be rewritten as

$\begin{matrix} F^{(2)}=F^{(1)}+(1-\frac{\kappa^{(1)}}{\kappa^{(2)}})G^{(1)}(-\ln r_{1}+\ln C) \\ G^{(2)}=\frac{\kappa^{(1)}}{\kappa^{(2)}}G^{(1)} \end{matrix}$ (S5)

1. When, the difference between and becomes infinitesimally small, and the two can be written in terms of a single function:

$F^{(1)}=F(r_{in}),F^{(2)}=F(r_{1})\approx F(r_{in})+h[\frac{dF(r)}{dr}]_{r=r_{in}}$ (S6)

Similar formulas hold for $G^{(1)}$ and $G^{(2)}$, and for all other $F^{(i)}s$ and $G^{(i)}s$. So the formulas hold:

$\begin{matrix} F^{(1)}=F(r_{in}),G^{(1)}=G(r_{in}) \\ F^{(2)}=F(r_{1})\approx F(r_{in})+h[\frac{dF(r)}{dr}]_{r=r_{in}},G^{(2)}=G(r_{1})\approx G(r_{in})+h[\frac{dG(r)}{dr}]_{r=r_{in}} \\ \frac{\kappa^{(1)}}{\kappa^{(2)}}=[\frac{\ln r_{in}-\ln C}{\ln(r_{in}+h)-\ln C}]^{\alpha}\approx1-\frac{\alpha h}{r_{1}\ln(r_{1}/C)} \\ r_{1}=r_{in}+h=r_{in}(1+\frac{h}{r_{in}}),\frac{1}{r_{1}}\approx\frac{1}{r_{in}}(1-\frac{h}{r_{in}}) \end{matrix}$(S7)

where F(r) and G(r) are two sufficiently smooth functions. The substitution of Eq. (S7) into Eq. (S5) and the application of $h\to0$ to the resultant equations lead to the following two simultaneous differential equations:

$\frac{dF(r)}{dr}=-\alpha\frac{G(r)}{r},\frac{dG(r)}{dr}=-\frac{\alpha}{\ln(r/C)}\frac{G(r)}{r}$ (S8)

1. All continuity conditions at the interfaces, in the form of 2(n-1) simultaneous algebraic equations, reduce to two simultaneous differential equations, the exact solution for the NGD is obtained as:

$T(r)=F(r)+G(r)(-\ln r+\ln C)$ (S9)

By solving the two simultaneous differential equations for and , the results are as:

$G(r)=-F_{0}(\ln\frac{r}{C})^{-\alpha}$ (S10)

and

$F(r)=\left\{\begin{matrix} G_{0}+\frac{\alpha F_{0}}{1-\alpha}(\ln\frac{r}{C})^{1-\alpha},\alpha\neq1 \\ G_{0}+F_{0}\ln(\ln\frac{r}{C}),\alpha=1 \end{matrix}\right.$ (S11)

The substitution of Eq.(S10) and Eq.(S11) into Eq.(S9) yields the temperature solution as follows:

$T(r)=\left\{\begin{matrix} G_{0}+\frac{F_{0}}{1-\alpha}(\ln\frac{r}{C})^{1-\alpha},\alpha\neq1 \\ G_{0}+F_{0}+F_{0}\ln(\ln\frac{r}{C}),\alpha=1 \end{matrix}\right.$ (S12)

For $\alpha\neq1$, when

$\begin{matrix} r=r_{in},T(r_{in})=G_{0}+\frac{F_{0}}{1-\alpha}(\ln\frac{r_{in}}{C})^{1-\alpha} \\ r=r_{out},T(r_{out})=G_{0}+\frac{F_{0}}{1-\alpha}(\ln\frac{r_{out}}{C})^{1-\alpha} \end{matrix}$ (S13)

For $\alpha=1$, when

$\begin{matrix} r=r_{in},T(r_{in})=G_{0}+F_{0}+F_{0}\ln(\ln\frac{r_{in}}{C}) \\ r=r_{out},T(r_{out})=G_{0}+F_{0}+F_{0}\ln(\ln\frac{r_{out}}{C}) \end{matrix}$ (S14)

Substitution of Eq. (S13) and Eq. (S14) into Eq. (S12), the finial temperature solution:

$\begin{matrix} T(r)=\left\{\begin{matrix} T(r_{in})+[T(r_{out})-T(r_{in})]\frac{\left[\ln(r/C)\right]^{1-\alpha}-\left[\ln(r_{in}/C)\right]^{1-\alpha}}{\left[\ln(r_{out}/C)\right]^{1-\alpha}-\left[\ln(r_{in}/C)\right]^{1-\alpha}},\alpha\neq1 \\ \\ T(r_{in})+[T(r_{out})-T(r_{in})]\frac{\ln\left[\ln(r/C)\right]-\ln\left[\ln(r_{in}/C)\right]}{\ln\left[\ln(r_{out}/C)\right]-\ln\left[\ln(r_{in}/C)\right]},\alpha=1 \end{matrix}\right. \\ \begin{matrix} & \end{matrix}=\left\{\begin{matrix} T(r_{in})+[T(r_{out})-T(r_{in})]\frac{\text{R}_{normal}\left(\text{r}\right)^{1-\alpha}-\left[\ln(r_{in}/C)/\ln(r_{out}/C)\right]^{1-\alpha}}{1-\left[\ln(r_{in}/C)/\ln(r_{out}/C)\right]^{1-\alpha}},\alpha\neq1 \\ \\ T(r_{in})+[T(r_{out})-T(r_{in})]\frac{\text{R}_{normal}\left(\text{r}\right)-\left[\ln(r_{in}/C)/\ln(r_{out}/C)\right]}{1-\left[\ln(r_{in}/C)/\ln(r_{out}/C)\right]},\alpha=1 \end{matrix}\right. \end{matrix}$(S15)

where C is a constants between 0 and $r_{in}$. In the figures, we choose the value of C as r_in_/e for the purpose of normalization.





Figure S1. (Color on-line) Besides the Nosé-Hoover method, the Langevin heat bathes are also used in MD simulation. (a) The temperature profiles of graphene disk along the radial direction with different temperatures. The outer radius of disk equals 13.89 nm. The symbols are MD simulation results and the lines are fitted lines based on analytical results in Eq. (S15). The fitting values of are 1.35±0.01, 1.11±0.01, 0.66±0.01, 0.51±0.01, and 0.46±0.01, corresponding to the temperature as 300, 600, 1000, 1500, and 2500 K, respectively. (b) The graded thermal conductivity of graphene disk with temperature from 300 K to 2500 K. For convenience of comparison, the values of thermal conductivity by Nosé-Hoover (as the same as shown in figure 2 (b)) are also shown here. The error bar is a combination of the error in the calculation of temperature gradient and the standard error of heat flux which is an average of 12 MD simulations with different initial conditions.
